# Gustatory sensitivity to amino acids in bumblebee mouthparts

**DOI:** 10.1098/rsos.250465

**Published:** 2025-05-07

**Authors:** Sergio Rossoni, Rachel H. Parkinson, Jeremy E. Niven, Elizabeth Nicholls

**Affiliations:** ^1^ Department of Ecology and Evolution, University of Sussex, Brighton, East Sussex, UK; ^2^ Department of Biology, University of Oxford, Oxford, Oxfordshire, UK

**Keywords:** taste, valine, lysine, protein, pollen, nectar, nutrition, pollination

## Abstract

Bees rely on amino acids from nectar and pollen for essential physiological functions. While nectar typically contains low (less than 1 mM) amino acid concentrations, levels in pollen are higher but variable (10–200 mM). Behavioural studies suggest bumblebees have preferences for specific amino acids but whether such preferences are mediated via gustatory mechanisms remains unclear. This study explores bumblebees’ (*Bombus terrestris*) gustatory sensitivity to two essential amino acids found in nectar and pollen, valine and lysine, using electrophysiological recordings from gustatory sensilla on their mouthparts. Valine elicited a concentration-dependent response from 0.1 mM, indicating that bumblebees could perceive valine at concentrations found naturally in nectar and pollen. By contrast, lysine failed to evoke a response across tested concentrations (0.1–500 mM). The absence of lysine detection raises questions about the specificity and diversity of amino acid-sensitive receptors in bumblebees. Bees responded to valine at lower concentrations than sucrose, suggesting comparatively higher sensitivity (EC_50_: 0.7 mM versus 3.91 mM for sucrose). Our findings indicate that bumblebees can evaluate the amino acid content of pollen and nectar using pre-ingestive cues, rather than relying on post-ingestive cues or feedback from their nestmates. Such sensory capabilities probably impact foraging strategies, with implications for plant–bee interactions and pollination.

## 1. Introduction

Bees’ ability to exploit floral resources efficiently depends upon their evaluation of the food rewards offered. Bees base their foraging decisions for nectar on parameters including sugar concentration [1], flow rate [2] and distance from the nest [3]. In addition to carbohydrates obtained from nectar, bees also require protein for maintenance, sexual maturation and larval development [4]. Amino acids are the building blocks of proteins and can be obtained from both pollen and nectar [5]. While highly variable between species and even individual plants [6,7], amino acids are typically found in pollen at 10−200 mM [8–11] and, although found at much lower concentrations in nectar (less than 1 mM), are still the most abundant nutrient after sugars [12–15].

Amino acids influence bee feeding and foraging choices in compound- and concentration-dependent ways [16–18]. Bees prefer to collect pollen with higher levels of amino acids [19], though some amino acids can increase nectar consumption at low concentrations but inhibit consumption at high concentrations, e.g. proline [20,21]. Bees also prefer nectars containing one or more essential amino acids (EAA) that cannot be synthesized and must be obtained from the diet [22], over those containing only non-essential amino acids (NEAA) [15,23].

Whether the effects of amino acids on bees’ dietary choices are mediated by pre-ingestive taste cues remains uncertain [4,15]. The apparent regulation of amino acid intake by bees at the individual and colony level could arise from post-ingestive processes or, in the case of social bees, feedback from nestmates or larvae. A behavioural study using chemo-tactile conditioning of the proboscis extension response (PER) [24] found that when presented to their antennae, bumblebees can distinguish some EAAs (e.g. lysine) and NEAAs (e.g. glutamate) from water but not others (e.g. valine and proline, respectively). Moreover, bees could not discriminate between different amino acids [24]. However, PER assays are unable to disentangle olfactory, gustatory and tactile cues; honeybees have been shown to smell certain amino acids at high concentrations, albeit beyond those found naturally in nectar [25]. To prevent potential stimulation of both olfactory and gustatory receptor neurons, gustatory sensilla can be stimulated in the proboscis, which is devoid of olfactory sensilla. Moreover, mouthparts are also more likely to contact floral rewards, especially in plants where nectar and pollen are secluded [26–29]. The galea is a particularly tractable part of the proboscis, upon which two morphologically distinct types of gustatory sensilla are found [30]. The longer, A-type sensilla contain four gustatory receptor neurons [30]. In bumblebees, only the response of these sensilla to a range of sugars has been examined [30,31]. In honeybees, electrophysiological recordings show that these sensilla respond to sucrose [30] as well as two NEAAs, glutamate and aspartate [32]. The receptor responsible for mediating this response to amino acids, *AmGr10*, shows amino acid-specific responses when expressed in cell lines [32], binding several EAAs (e.g. lysine) and NEAAs (e.g. glutamate and aspartate), but exhibiting no response to other amino acids, such as the EAA valine, and other sweet or bitter compounds.

Here, we conducted the first electrophysiological recordings of gustatory responses to amino acids from bumblebees, testing whether the buff-tailed bumblebee (*Bombus terrestris*) can detect amino acids pre-ingestively via gustatory sensilla. Our choice of amino acids was motivated by (i) behavioural evidence from *B. terrestris* that shows lysine is perceived when contacted by the antenna, whereas valine is not [24]; and (ii) evidence from electrophysiology and imaging that shows lysine is detected by the honeybee *AmGr10* gustatory receptor, whereas valine is not [32]. Moreover, lysine and valine are EAAs both of which are found in floral nectar and pollen of a variety of plant species [6–10,12,15,33–39]. Thus, by testing with lysine and valine we expected to elicit a differential response in galeal gustatory receptor neurons, a concentration-dependent response to lysine and a weak response or no response to valine. This would both demonstrate the presence of a gustatory response to an amino acid in bumblebees and provide evidence of the specificity of this response. Therefore, we presented the gustatory sensilla on the bumblebee galea with valine and lysine across a range of concentrations (0.1−500 mM) encompassing amino acid concentrations found in both types of floral reward [11,12]. Our results demonstrate that bumblebee galeal gustatory receptor neurons can differentially respond to amino acids in a concentration-dependent manner but do so to valine and not lysine.

## 2. Material and methods

### 2.1. Animal husbandry and selection

Buff-tailed bumblebee (*Bombus terrestris audax*) colonies were obtained from Biobest (Westerlo, Belgium) (*n* = 2) and Koppert (Berkel en Rodenrijs, The Netherlands) (*n* = 1). Colonies were maintained at the University of Sussex, UK, housed either within a flight cage (73 × 73 × 65 cm) or connected to a feeding arena (40 × 40 × 35 cm) via a corridor (4 × 4 × 26 cm). Bees had ad libitum access to a nectar substitute (Biogluc, Biobest, Westerlo, Belgium) via both ground and suspended feeders, and finely ground honeybee collected pollen (Agralan, Swindon, UK), presented on chenille stems placed inside white plastic cups. Nectar was replenished as needed and pollen changed daily. Workers observed collecting pollen from the feeders were marked on the thorax with a small dot of white enamel paint (Humbrol, Hornby Hobbies Limited, Margate, UK). Only individuals observed collecting pollen at least once were used in testing (*n* = 27).

### 2.2. Animal preparation and restraint

Bumblebees (*n* = 27) were caught in the feeding arena and left at 4°C overnight to immobilize them. On the day of recording, bees were inserted in small plastic tubes and harnessed using small strips of Parafilm M (American National Can, Greenwich, CT, USA). Bees were then transferred to a plate of sealing wax beneath a microscope (Nikon AZ100, Tokyo, Japan). Using wax, the head, antennae and front legs of the bumblebee were immobilized, and the glossa and labial palps were manually extended and immobilized to the plastic tube. The galeae were rinsed in ultrapure water and dried with QL100 filter paper (Fisherbrand, Fisher Scientific, Loughborough, UK), and subsequently extended and fixed to the plastic tube using small strips of Parafilm to prevent heat damage.

### 2.3. Electrophysiological recordings

**Figure 1.**
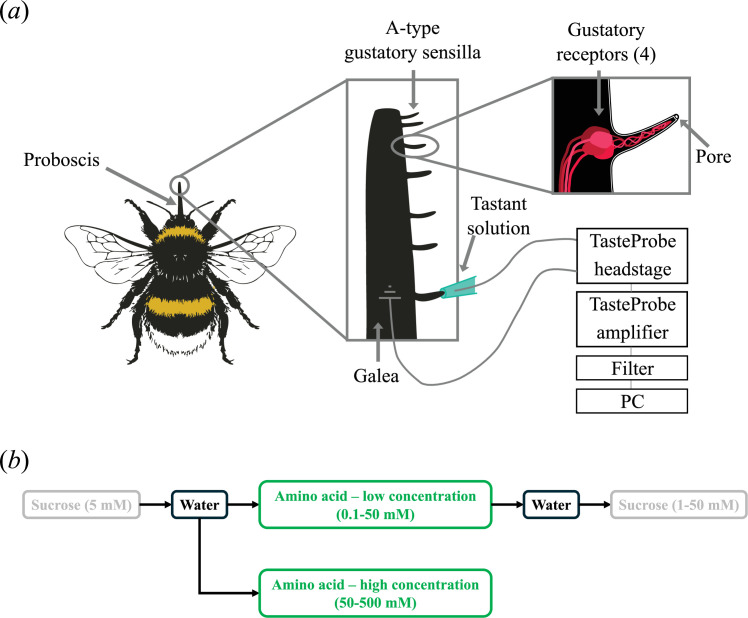
(*a*) The experimental set-up for recording from gustatory sensilla on the bumblebee galea. (*b*) The stimulation protocol.

Extracellular tip recordings (figure 1*a*) were performed on A-type sensilla chaetica on the left galea [30]. A 25 μm tungsten wire (Alfa Aesar, Ward Hill, MA, USA) was inserted into the galea at the proximal end and pushed gently down to approximately 1 mm from the sensilla from which the recording was to be made. This wire was used as the reference electrode. The recording electrode was a 250 μm silver/silver chloride wire, placed inside a borosilicate glass capillary (1 × 0.58 × 100 mm, OD × ID × L); Harvard apparatus, Holliston, MA, USA) pulled on a P-97 micropipette puller (Sutter Instrument Co., Novato, CA, USA) to a tip diameter of approximately 20–50 μm to fit comfortably over A-type gustatory sensilla on the galea (*n* = 124) without deflecting the hair. Capillaries were filled with tastant solutions (see below) with no added electrolytes. The capillary was moved close to the galea using an LBM-7 manipulator (Scientifica, Uckfield, UK) and contact with the apical pore was made using an MO-203 micromanipulator (Narishige, Tokyo, Japan), mounted on the LBM-7.

Electrical signals, which represent the ensemble activity of the four gustatory receptor neurons (GRNs) innervating each sensillum (figure 1*a*), were recorded through the tastant solutions. The electrodes were connected to a TasteProbe [40] headstage and TasteProbe DTP-02 10× amplifier (Syntech, Buchenbach, Germany). Recordings were made for 20 s with a 1 Hz high-pass filter. The signal was further amplified with 5× gain and band-pass filtered between 10 Hz and 10 kHz (LHBF-48X filter-amplifier, npi electronic GmbH, Tamm, Germany). Recordings were digitized via a CED micro1401-3 analogue-to-digital converter (Cambridge Electronic Design Ltd, Cambridge, UK) and acquired using CED Spike2 software (10.09) at 20 kHz.

### 2.4. Tastant stimulation and protocol

Gustatory sensilla on the galeae (*n* = 3–6 sensilla per bee) were stimulated with aqueous solutions of D(+)-sucrose (Fisher Scientific, Loughborough, UK), L-valine (Thermo Fisher Scientific, Heysham, UK) or L-lysine (Thermo Fisher Scientific, Waltham, MA, USA), with no added electrolytes. For solutions with valine concentrations above 100 mM, hot (between 60 and 90°C) water was used to aid valine dissolution. Syringes filled with tastant solutions were prepared in advance and stored at −10°C. Prior to the electrophysiological experiments, the syringes were thawed, and stored at 4°C. The capillary used for stimulation was filled immediately prior to use, to minimize water evaporation affecting solution concentration. Bees were allocated to one of two experimental groups, one stimulated with low amino acid concentrations and one with high concentrations (figure 1*b*), to prevent slow adaptation over the course of the experiment. In both conditions, gustatory sensilla were tested initially with 5 mM sucrose and water, to ensure that sucrose sensitivity was comparable between GRN ensembles in all conditions. Bees in the low-concentration group were exposed to increasing concentrations of either lysine or valine from 0.1 to 50 mM. Then, bees in this group were tested with increasing concentrations of sucrose from 1 to 50 mM [30]. The high-concentration group was exposed to increasing concentrations of either lysine or valine from 50 to 500 mM. At least 3 min elapsed between exposure to different tastants and concentrations to reduce possible adaptation. Temperature was monitored every 30 s throughout the recording, using an automated temperature logger (EasyLog-USB-2-LCD, Lascar Electronics, Whiteparish, UK).

### 2.5. Data processing

Individual recordings were imported into Matlab (R2024a, MathWorks Inc., Natick, MA, USA) for offline analysis based on [31]. Recordings were trimmed either using contact artefacts, or by the 20 s timestamp acquired from the amplifier, whichever was shortest; the entire trimmed length was used for data extraction. Filtered recordings were then created using a band-pass second-order Butterworth filter between 100 and 1000 Hz. Using these copies, a threshold was then selected manually for each recording to identify spikes. The peak of the spikes above the threshold was used to acquire 4 ms waveforms from the raw signal. The waveforms in each recording were inspected. Four measures were used to distinguish spikes and artefacts: maximum voltage reached, minimum voltage reached, waveform amplitude and waveform half-width. Individual waveforms with unusual or irregular shapes were considered movement artefacts and removed. Spike frequency was calculated as the total number of spikes in the recording divided by the duration after trimming [41]. To describe the change in spike frequency to increasing stimulation of tastants, we fitted a sigmoid using the method of nonlinear least squares in the ‘stats’ package using R studio (4.4.1 R software [42]). For the final figures, electrophysiological recordings were smoothed with a 15 Hz high-pass Butterworth filter and taken from 20 ms after trimming.

### 2.6. Statistical analysis

Statistical tests were implemented using R studio software, version 2024.04.2, and R software, version 4.4.1 [42]. Homogeneity of variance was tested using Levene’s test in the ‘car’ package. Two datasets with Gaussian sampling distribution and homogeneous variance were compared using a Student’s *t*‐test in the ‘stats’ package. A generalized linear mixed effects model (GLMM) implemented in the ‘lmerTest’ package was used to test whether tastant concentrations affected spike frequency: log_10_(spike frequency) ~ log_10_[amino acid] + (1|temperature) + (1|sensillum). A GLMM was also used to compare the concentration dependency between valine and sucrose: log_10_(spike frequency) ~ substance ***∗*** log_10_[tastant] + (1|temperature) + (1|sensillum). To avoid mathematical infinity when using transformed data at 0 mM or 0 Hz, 1 was added to all spike frequencies and tastant concentrations prior to logarithmic transformation. Where relevant, GLMMs with significant predictors were followed by post hoc pairwise comparisons with Hommel’s adjusted *p-*values in the ‘stats’ package.

## 3. Results

**Figure 2.**
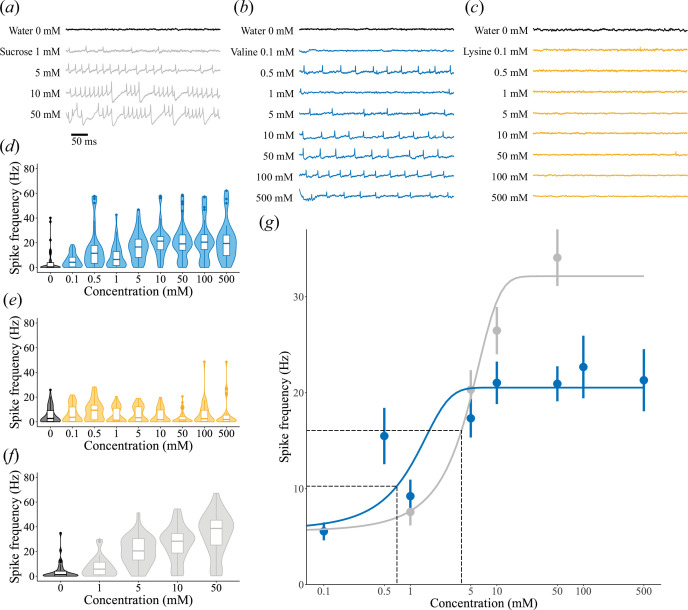
Gustatory neurons in sensilla show concentration dependency in response to valine and sucrose but not to lysine. (*a*) Example recording of one gustatory sensillum in response to water (black) and increasing concentrations of sucrose (grey). (*b*) As in *a* but for valine (blue). (*c*) As in *a* but for lysine (yellow). (*d*) The spike rate (Hz; median and interquartile range (IQR)) of gustatory neurons over approximately 20 s in response to water (black) and increasing concentrations of valine (blue). (*e*) As in *d* but for lysine (yellow). (*f*) As in *d* but for sucrose (grey). (*g*) Sigmoid fits to the spike rates for valine (blue) and sucrose (grey). Dashed lines indicate the half maximal effective concentration (EC_50_) and its corresponding spike frequency.

We observed reliable activation of the GRNs in response to sucrose (figure 2*a*). Higher sucrose concentrations (greater than 25 mM) typically triggered burst-like activity in the GRNs (figure 2*a*) [30]. GRNs also responded to stimulation with the amino acid valine between 0.1 and 500 mM (figure 2*b*), though no burst-like activity was recorded during valine stimulation, even at high (500 mM) concentrations. By contrast, little activity was seen during stimulation with the amino acid lysine, even at high (500 mM) concentrations (figure 2*c*).

Valine concentration was a significant predictor of GRN spike frequency (*n* = 59, GLMM: *t* = 13.7, coefficient estimate 0.349, standard error (s.e.) 0.026, *p* < 0.001), indicating that sensitivity to valine was concentration dependent (figure 2*d*). While stimulation with 0.1 mM valine did not significantly increase the spike frequency above baseline (*n* = 93, pairwise comparison post hoc, *p* = 0.054), 0.5 mM valine significantly increased the spike frequency (*n* = 93, *p* < 0.001), which plateaued at a concentration of 5 mM (1 versus 5 mM post hoc: *n* = 68, *p* = 0.025, 5 versus 10 mM post hoc: *n* = 93, *p* = 0.980). By contrast, lysine concentration did not significantly predict spike frequency (figure 2*e* *; n* = 65, GLMM: *t* = −1.07, coefficient estimate −0.022, s.e. 0.021, *p* = 0.285). The responses to 5 mM sucrose presented to sensilla subsequently exposed to valine or lysine were not significantly different (*n* = 69, Student’s *t*‐test, *t*(67.0) = 0.894, *p* = 0.375), indicating that the two groups of bees did not differ in their responses to sugars.

We compared the responses with valine (0.1−50 mM) and sucrose (1–50 mM, figure 2*f*) from sensilla exposed to both tastants. There was a significant interaction between the tastant (sucrose or valine) and concentration (*n* = 34, GLMM: *t* = −3.38, coefficient estimate −0.212 for valine, s.e. 0.063, *p* < 0.001): increasing valine concentrations evoked lower spike frequencies than increasing sucrose concentrations (figure 2*g*). The sigmoid fitted to the frequency response, *f*, of GRNs to increasing sucrose concentrations, *c*, was *f* = 32.1/(1+e^−(c-3.91)0.399^). The sigmoid fitted to the frequency response of GRNs stimulated with increasing valine concentrations was *f* = 20.5/(1+e^−(c-0.699)1.34^). Sucrose had a higher frequency plateau (*f*_max_ = 32.1 Hz) and half-maximal effective concentration (EC_50_ = 3.91 mM) than valine (*f*_max_ = 20.5 Hz, EC_50_ = 0.699 mM). Thus, gustatory sensilla responded to valine with a lower frequency plateau, reached at lower concentrations, compared with sucrose.

## 4. Discussion

We demonstrate that bumblebees can perceive the EAA valine pre-ingestively via gustatory sensilla on their mouthparts, specifically the galea. This response was dose-dependent, reaching an asymptote at 5 mM. By contrast, the response of sensilla to the EAA lysine was not significantly different from water, suggesting bumblebees are unable to perceive lysine pre-ingestively via galeal A-type gustatory sensilla between 0.1 and 500 mM. An increase in spike rate was first elicited for 0.5 mM valine, and the EC_50_ (half maximal effective concentration) was 0.7 mM, suggesting that bumblebees are sensitive to lower concentrations of valine than sucrose (EC_50_ = 3.91 mM). The absence of burst-like firing in response to valine, even at high concentrations, suggests differing mechanisms for valine and sucrose detection, as burst firing has been previously hypothesized to prevent sensory adaptation to high sucrose concentrations [30]. Thus, bumblebees’ galeal GRNs are both specific and sensitive in their responses to at least one EAA, valine.

Other studies have also observed heterogeneous responses of bees to amino acids, at the molecular, electrophysiological and behavioural level [20,21,24,32]. Our results contrast with previous behavioural observations. When amino acids were presented to the antennae in a differential PER assay [24], bumblebees could not distinguish valine (85 mM) from water but could distinguish lysine (8.5−170 mM) within the concentration ranges we used to stimulate sensilla. While the PER method does not distinguish between olfactory, tactile and gustatory cues, which might also contribute to amino acid perception, such a discrepancy could also arise from differential expression of receptors between the antenna and mouthparts [43] or from age and/or experience-related differences in receptor expression between the bees tested in the two studies [44]. We selected bumblebee foragers observed collecting pollen, whereas Ruedenauer *et al*. [24] selected workers, not specifying whether they were foraging or in-hive bees.

One other study has performed single sensillum recordings in response to amino acids from the mouthparts of bees; Lim *et al*. [32] measured the sensitivity of honeybee (*Apis mellifera*) galeal GRNs to two NEAAs, glutamate and aspartate, finding a dose-dependent response to both with an increasing response between 50 and 200 mM. Voltage-clamp recordings and calcium imaging of expressed honeybee *AmGr10* gustatory receptors (GRs) demonstrated a response to multiple amino acids to differing degrees but not sweet or bitter substances [32]. Expressed *AmGr10* exhibited sensitivity to lysine but not valine, again contrasting with our findings from bumblebee mouthpart recordings, which showed the opposite sensitivity. There could be multiple explanations for this difference. Although *Bombus terrestris* does possess a GR (XP_02718455) homologous to *AmGr10* [32], its ligand specificity may differ so that it binds valine instead of lysine. Another possibility is that the responses to valine we recorded are mediated by a different receptor. Were this true, *AmGr10* may be absent or could bind other compounds in bumblebee mouthparts. It is worth noting that *AmGr10* is more highly expressed in the antennae of honeybees than in their proboscis. Interestingly, Lim *et al.* [32] observed that sensitivity to both lysine and valine could be enhanced by the addition of purine ribonucleotides that are also found in pollen and may therefore play a role in enhancing nutrient perception by bees.

Much of our understanding of gustatory perception in insects, particularly beyond behavioural assays, derives from studies on flies. Their gustatory responses to amino acids are also compound- and concentration-dependent [45,46] and vary according to sensilla type [47]. Several mechanisms are thought to be involved in amino acid perception at the mouthparts, involving both sweet-sensing [47–49] and bitter-sensing [47] GRNs. Furthermore, amino acid perception relies on both metabotropic GRs and ionotropic receptors (IRs) [46,47]. *Drosophila melanogaster* labellar cells show a biphasic response to some amino acids: lysine elicits a weakly attractive response via the sweet-sensing GRNs at low concentrations, whereas high concentrations are aversive and activate the IRs in bitter-sensing GRNs [46]. Valine by contrast, elicited a response in bitter-sensing GRNs at both low and high concentrations. For both valine and lysine perception, IRs were required. The *Drosophila melanogaster* genome contains 66 IR genes, whereas the *Bombus terrestris* genome contains just 21 IR genes [50]. Although some IRs have been shown to be conserved between *D. melanogaster* and *A. mellifera* [51], only antennal IRs have been considered and no studies have examined IR function in bee gustation. Some flies express valine-gated IRs in their labellar sweet taste cells [48], raising the possibility that valine is similarly detected via sugar-sensitive GRNs or a dedicated GRN sensitive to some, but not all, amino acids. Combined with the absence of lysine response in bumblebee mouthpart sensilla, this suggests further work is needed to conclude whether bumblebees truly possess dedicated amino acid receptor neurons, as is suspected of honeybees [32].

This newly demonstrated capacity for pre-ingestive amino acid perception provides a potential mechanism for bumblebees to rapidly assess floral rewards while foraging, based on amino acid as well as sugar content, which may guide their foraging choices and have significant implications for bee–flower interactions and pollination. Bumblebees responded to valine at 0.5 mM, meaning they may be able to perceive it in nectar, where concentrations rarely exceed 1 mM [12–15], as well as pollen, where amino acids are found, both free and bound in proteins, between 10 and 200 mM [8–11]. It is already known that bees have dose-dependent feeding preferences for amino acids when presented in sugar solutions, and so it is likely that amino acid concentrations, which vary considerably between the nectar and pollen of different plant species or even between plants within the same species [6,7], could impact individual foraging choices in real time, without the need for post-ingestive cues or feedback from nestmates on return to the colony [52]. While the mouthparts of bumblebees undoubtedly come into contact with nectar during feeding, and with pollen during flower handling and the addition of nectar for pollen packing into the corbiculae [29], there remain open questions regarding the opportunities for adult bees to sample nutritional cues from pollen pre-ingestively, since the majority of free amino acids are found inside the pollen grain [5]. Evidence from other insects, such as butterflies, shows that pollen grains can be lysed at the mouthparts through osmotic and mechanical processes to release the nutritional contents inside [52,53]. If these mechanisms also occur at the bumblebee mouthparts, then bees would also be able to detect the presence and concentration of certain amino acids during pollen collection. Further work is also needed to determine whether bees evaluate the nutritional quality of pollen via the presence or specific concentration of a single common amino acid or rather use a combination of amino acids (i.e. those most found across different pollen species) as a more reliable gustatory cue.

## Data Availability

Data are available for review on Dryad data repository [41].
